# How facilitators use healthcare students’ mistakes to promote reflections and discussions during simulation debriefings

**DOI:** 10.1186/s41077-026-00412-3

**Published:** 2026-02-05

**Authors:** Wenche Lervik, Mads Solberg, Astrid Camilla Wiig, Helen Berg

**Affiliations:** 1 Department of Health Sciences in Ålesund, The Norwegian University of Science and Technology, Ålesund, Norway; 2 Department of Educational Science, University of South-Eastern Norway, Porsgrunn, Norway

**Keywords:** Simulation-based training, Facilitator, Mistakes, Debriefing, Feedback, Health education, Education and training, Psychological stress

## Abstract

**Background:**

Simulation-based training is increasingly used in healthcare education. It allows students to practice in realistic environments using high-fidelity patient simulators and to engage with complex or rare diagnoses without risking harm to real patients. During the debriefing phase, students and facilitators reflect on actions taken and discuss what went well and what could be improved in future simulation sessions. To engage in productive reflection, students must be aware of their mistakes and performance gaps. Although the literature emphasizes the importance of providing both positive and corrective feedback, many facilitators struggle to deliver negative feedback due to concerns about hurting students’ feelings or damaging their relationships. This study explores how facilitators use nursing students’ mistakes to prompt reflection and discussion during healthcare simulation debriefings.

**Method:**

This was a qualitative video-supported observational study. 17 facilitators and 89 students from three universities in Norway participated in mandatory simulation-based training in their second year of education. The framework of Heath, Hindmarsh, and Luff (2010) inspired video analysis. The units of data consisted of verbal utterances, bodily conduct, gaze, and facial expressions, as noted through observations of participants’ turns at talk. We complemented this by using thematic analysis inspired by Braun & Clarke of selected video transcripts to support and deepen the analysis.

**Results:**

The facilitators who elicited the most reflections and discussions among the students during debriefings consistently employed five communication elements: inquiries, positive feedback, hints and cues, suppressions, and summarizing.

**Conclusion:**

The main findings provide new insights into facilitators’ actions when eliciting student reflections and discussions. Many structured debriefing frameworks today include inquiries, positive feedback, and hints and cues. This analysis identified additional communication elements not previously recognized in the debriefing literature, namely suppressions and the use of summaries. Few studies have examined the facilitators’ actions during debriefings to elicit student reflection and discussion, particularly through naturalistic observation of real-world practice. Further research is needed to expand our understanding of these interactional dynamics and the situated strategies employed by facilitators.

**Supplementary Information:**

The online version contains supplementary material available at 10.1186/s41077-026-00412-3.

## Introduction

Simulation-based training offers a safe environment for students to practice clinical skills and learn from mistakes without risking patient safety [1]. Mistakes are essential to learning, as feedback on both successes and failures enhances understanding [2, 3]. In our study, mistakes are defined as nursing students’ actions or behaviours that could have had clinical consequences for the patient [4].

Mistakes should be seen as learning opportunities, with feedback focused on the situation rather than the student’s personal value [2, 3]. However, giving and receiving feedback can be difficult. Facilitators may worry that feedback could damage their relationship with students or provoke defensiveness, discouraging future participation. This is known as the “task-versus-relationship” dilemma [5].

Simulation-based training is a standard method in healthcare education, structured around briefing, scenario, and debriefing phases [6]. During the briefing, facilitators outline the scenario and learning goals. Students then engage in a realistic patient case using a high-fidelity simulator. In the debriefing, they reflect on their actions, identify challenges, and discuss ways to improve to reinforce learning [6].

Debriefing and feedback are key to adult learning, which emphasizes learning through experience and reflection [7]. Cheng et al. [8] describe debriefing as a two-way, reflective dialogue among team members. It can be led by a facilitator or guided by students themselves [6], though the gold standard is facilitator-led debriefing that encourages reflection on strengths and areas for growth [9].

Facilitators provide feedback on performance, highlighting what went well and what could be improved [7, 10]. Feedback is defined as “information about performance provided to simulation participants with the intent to modify thinking and/or behaviours to facilitate learning and improve future performance” [11].

According to the INACSL Standards Committee, debriefing should be led by someone skilled in the process [12, 13]. Effective debriefing relies heavily on the facilitator’s ability to guide reflective discussions [14]. Teachers acting as facilitators must have the education, motivation, and skills needed to support students in achieving the learning objectives [6, 15]. Facilitators are also encouraged to use structured frameworks to guide debriefing sessions [12]. These frameworks, often including distinct phases such as reaction, analysis, and summary, help organize discussions and promote student reflection [16]. While many frameworks exist, there is no consensus about which is preferred [7].

Allowing students to reflect on their mistakes enhances the quality and meaning of the debrief [17]. In contrast, when reflection is limited, students may develop poor clinical reasoning and make mistakes in clinical practice [18]. In simulation literature, “effective” and “ineffective” debriefings typically refer to how the facilitator conducts the session, rather than the actual effect on learning outcomes [19].

Providing constructive feedback, both positive and negative, is nonetheless considered essential [13]. When feedback on actual mistakes made by students is withheld, valuable learning opportunities may be lost during debriefing [20].

Few studies have examined how facilitators encourage reflection on students’ mistakes during debriefing. Lymer and Sjöblom [21] noted that while many quantitative studies focus on measuring learning outcomes, the details of debriefing interactions remain largely unexplored. Moreover, debriefing frameworks vary in how they guide facilitators, reflecting differences in practice [22–24]. Lervik et al. [25] explored how experienced facilitators view students’ mistakes as learning opportunities. These facilitators, with clinical backgrounds and formal training, used diverse approaches; some gave direct feedback, others preferred indirect methods, and a few avoided addressing mistakes altogether, assuming students were already aware.

Because of this variation, we need to better understand what skilled facilitators do to promote reflection and discussion. By examining debrief interactions, we can uncover what facilitators do to support student reflection and learning. This study therefore investigates how facilitators use students’ mistakes to foster reflection, drawing on naturalistic data from simulation debriefings.

## Methods

### Study design

This qualitative video-supported observation study is grounded in an interactional research paradigm, which views meaning as co-constructed through social interaction and emphasises the naturalistic analysis of talk and embodied conduct in situated learning contexts [26].

### Participants and recruitment

We contacted five nursing bachelors’ program directors in Norway, using existing connections within our research team to facilitate recruitment. Three of the five universities agreed to participate.

All facilitators were experienced nurses with completed facilitator courses. Their teaching experiences ranged from 2 to 26 years, and their experiences as facilitators from 6 to 11 years (Two had previous experience as facilitators from the clinic). All students were in their second year of education, both before and after their second clinical practice. Most were in their early twenties, and there were 89 women and 18 men. This was their first experience with a high-fidelity simulation.

All participants provided informed consent, based on verbal and written information, about the intention to observe and record the simulation sessions for analysis and publication. The information provided included the study’s purpose, duration, data handling, anonymization, and the right to withdraw at any time without explanation. Ethical approval was obtained – see Declaration for details.

In one university, 11 facilitators and 45 students consented to participate. In the second, two facilitators and 18 students consented, and in the third, four facilitators and 26 students agreed to participate. Overall, 17 facilitators and 89 students from the three universities provided written consent to participate in the study (Table 1).

**Table 1:** Overview of facilitators and students from each of the three universities (*N* = 106)

University	Facilitator	Students	Total
1	11	45	56
2	2	18	20
3	4	26	30
Total	17	89	106

### Setting and data collection

During autumn 2022 and spring 2023, the first author observed advanced simulation-based training sessions for nursing students at the three participating universities. A total of 36 sessions were video-recorded, each lasting 1.5–2.5 h, for approximately 70 h of video-recordings. Up to three tripod-mounted cameras and an audio recorder were used, depending on the setup, to capture both video and sound. While briefings and scenarios were recorded for context, the debriefing sessions served as the primary data for this study.

During each session, the first author kept detailed observation logs inspired by Heath, Hindmarsh, and Luff [26]. These included date, time, team composition, facilitator, scenario, learning objectives, personal reflections, and timestamps of key events. The logs supported the analysis process when systematically cataloguing all video recordings.

The training engaged nursing students in role-play scenarios using advanced patient simulators to simulate diagnoses such as cardiac arrest, chronic obstructive pulmonary disease (COPD), hypoglycemia, post-operative bleeding, and bronchiolitis. Students assumed roles as either nurses, relatives, or observers in each scenario. Observers monitored and documented how active students’ actions aligned with predefined learning objectives.

In all simulation sessions, students were assigned to different roles. Nurse 1 (N1) served as the lead nurse, assuming primary responsibility for the patient, including communicating with the doctor and making decisions regarding interventions and medications. Nurse 2 (N2) and, occasionally, Nurse 3 (N3) assisted by taking notes and performing vital signs, including heart rate, blood pressure, respiratory rate, and oxygen saturation. Occasionally, another student role-played the patient’s relative. The remaining students were Observers (O1–O9), each tasked with noting how the active students handled the various situations during the scenarios in accordance with the learning objectives. An operator, seated in an adjacent room, looked through a one-way window and controlled the simulator’s clinical parameters, acting as the “patient’s” voice and the responding doctor. A facilitator (Fac) accompanied the students throughout the simulations.

Key learning objectives focused on clinical assessment and management using the ABCDE approach (Airway, Breathing, Circulation, Disability, and Exposure), a systematic method for evaluating acutely unwell patients. Students also applied the NEWS (National Early Warning Score) scheme and practiced structured communication using the ISBAR (Identification, Situation, Background, Assessment, Recommendation) tool. Additional objectives emphasized teamwork, leadership, and safe medication handling.

All three universities followed a consistent structure for their simulations: a 5–10-minute briefing introducing the case and learning objectives, followed by a 10–15-minute scenario involving active role-play with 2 to 4 students and 3 to 9 students observing. The simulation was terminated with a 30–40-minute facilitator-led debriefing, during which all students were present.

The four scenarios outlined describe the case, the learning objective, the mistakes, and the roles of the active participants and observers. Below is a table showing the varying cases (Table 2).

**Table 2:** Overview of the four cases

Situation	Case	Learning objective	Mistakes	Active roles	Observers
1	A five-week-old baby was admitted to the children’s department due to labored respiration. The tentative diagnosis was bronchiolitis. When the baby’s condition deteriorated, N3 called the doctor.	Using the ISBAR communication tool.	N3 failed to use the ISBAR tool when calling the doctor.	N1 (responsible for the patient), N2 (first assistant), and N3 (second assistant); in addition, another student role-played the baby’s mother.	O1-O6 observed and noted how the active students achieved their learning objectives.
2	A 76-year-old man who had undergone surgery on the left hip last night. Now, his condition was deteriorating with a high respiration rate, a high heart rate, low blood pressure, and low SPO2. He was pale and complaining about pain in the operating area.	Observe and apply the ABCDE assessment framework.	N1 and N2 measured the patient’s vital values but did not notice the deteriorating condition due to post-operative bleeding.	N (responsible for the patient) and N2 (assistant).	O1 to O8 observed and wrote notes about how the active students managed their learning objectives.
3	A young woman, recently diagnosed with type 1 diabetes, was admitted to the hospital this morning due to hypoglycemia. Her blood glucose level was 1,8 mmol/L. She refused to eat or drink and eventually lost consciousness. The physician prescribed intravenous glucose, which the students were instructed to administer.	Safe medication handling.	N1 and N2 did not manage medication according to guidelines. The double-check was poorly performed.	N1 (responsible for the patient, talked with the physician, and prepared the medicine), and N2 (assistant, performed the double-check of the medicine).	O1-O9 observed and wrote notes about how the active students managed their learning objectives.
4	A 70-year-old man with COPD grade 3 was admitted to the hospital due to an exacerbation. The patient had a respiratory rate of 25/min, SPO2 of 85%, and a heart rate of 120/min. The patient was uncomfortable and had lost his O2 supply. During the debriefing, the team discussed the learning objective of observing and applying the ABCDE systematics.	Observe and apply the ABCDE assessment framework.	N1 and N2 did not prioritise their actions according to the ABCDE assessment framework.	N1 (responsible for the patient) and N2 (assistant).	O1-O6 observed and noted how the active students achieved their learning objectives.

### Research team and reflexivity

The research team consisted of WL, MS, ACW, and HB. WL and HB are registered nurses with clinical experience and familiarity with simulation-based teaching and debriefing, and HB has published simulation-based healthcare training using both quantitative and qualitative designs. WL, a PhD student focusing on debriefing practices, brought insights into facilitation and student learning but was new to video-based observation. MS is an anthropologist with experience in ethnographic studies of simulation-based practice that foreground social interaction. ACW, an education researcher, contributed expertise in observational studies and simulation pedagogy. Collectively, these backgrounds informed how we interpreted the interactional dynamics and organizational context of the debriefings.

### Analysis

Video analysis was inspired by the framework of Heath, Hindmarsh, and Luff [26]. Video recordings of simulation debriefings served as the foundational data for the qualitative interaction analysis. Our units of analysis consisted of verbal utterances, bodily conduct, gaze, and facial expressions, as noted through observations of participants’ turns at talk [27]. This was complemented by a thematic analysis of selected video transcripts to support and deepen the analysis. This helped identify recurring patterns and supported the abstraction of themes across the dataset.

In a preliminary review, the first author systematically catalogued all video recordings on an encrypted hard drive to facilitate access to relevant recordings in subsequent analysis. Data were organized in files and subfiles detailing the university, exact dates, specific simulation cases, session types (briefing, scenario, debriefing), and camera positioning. The observation logs were stored in a secure cabinet at the Norwegian University of Science and Technology (NTNU).

In the subsequent phase of the analysis, the first author, an experienced intensive care nurse and a trained facilitator, systematically re-examined the simulation catalogue to identify: a) feedback situations involving verbal participation from both facilitators and students,

b) instances where facilitators elicited students’ reflections on mistakes; and c) discussions addressing misunderstandings, mistakes in clinical observations and procedures, inadequate communication and collaboration, and unsafe medication handling. All 36 simulation sessions were reviewed in detail, with the observation logs used to contextualize the analysis.

The authors jointly selected 10 debriefing situations for further in-depth analysis. The remaining 26 situations were excluded because the facilitators did not address mistakes or elicit reflective dialogue among the students. The first author transcribed fragments from the 10 selected situations verbatim and organized them into an analytic table, with columns detailing team composition, case, learning objectives, student roles, facilitator feedback, and student responses.

Further analysis of the 10 situations showed that some facilitators adopted a didactic approach, focusing on teaching rather than facilitating student reflection. Some debriefings involved more talk from the facilitator than actively elicited student reflections. In several debriefings, the facilitators confirmed students’ actions or told them what they could have done differently. Individual students sometimes dominated conversations, limiting broader engagement and deviating from best practices that encourage group participation. Ultimately, the facilitators successfully fostered reflection and discussion in 4 of the 10 situations.

The first author transcribed the talk in these four debriefing situations verbatim. These transcripts were analysed and further examined to deepen our understanding. Drawing on Braun and Clarke’s [28], thematic analysis was employed; these four transcribed debriefing situations were coded using NVivo 14.0 software to support qualitative data extraction. Initial coding generated 52 codes, which were collated into potential themes. Some codes were merged, reclassified, or omitted. Related codes were grouped into thematic clusters and iteratively discussed among the co-authors, leading to the subsequent refinement of the themes. A thematic map was developed to visualize the relationships between themes and codes.

The first and last authors jointly validated the final themes for the subsequent analysis. Five key communication elements were identified across the four situations: inquiries, positive feedback, hints and cues, suppressions, and summaries. The facilitators consistently used these elements to elicit students’ reflections on mistakes during debriefings.

In the next section, the five key communication elements are presented and discussed, drawing on the research literature and excerpts from the four case situations.

## Results

During the analysis, five themes developed: inquiries, positive feedback, cues/hints, suppressions, and summaries. The first three elements are well-established in the debriefing literature and explicitly incorporated into various debriefing frameworks [7, 29–31]. We therefore provide a brief introduction to them here and revisit them in the discussion section. In contrast, suppression and the use of multiple summaries are not addressed in the existing debriefing literature, indicating a novel contribution to our current understanding of debriefing practice.

### Inquiries

The communication element of inquiries describes how the facilitators used different question-asking strategies when initiating and approaching a topic to elicit students’ reflections and discussions. Typically, open-ended questions were frequently used, but there were also elements of advocacy inquiries and circular questions.

#### Open-ended questions

Simulation research and the best-practice literature have argued that open-ended questions provoke learner self-reflection and self-assessment [32, 33]. Asking open-ended questions is a well-established method for eliciting more detailed responses and encouraging students to elaborate on the subject matter [2]. It is recommended that reflective debriefings begin with open-ended questions, allowing the facilitator to provide space for students to express their immediate emotional reactions to the scenario. All the stages in the debriefing are followed by guidance and feedback from the facilitator [34, 35], and the facilitator should avoid closed-ended or “yes/no” questions, which seldom encourage students’ reflection [34]. A way to pose open-ended questions is to initiate them with words like “what,” “when,” “how,” and “why,” which might open the door to critical thinking and elaboration of clinical reasoning [34].

In the following excerpt, we present a situation in which the facilitator uses open-ended questions to ask N3 how she assessed the telephone conversation with the doctor during the episode when the five-week-old baby’s breathing deteriorated (see Supplementary file 1, case 1 for details) (Table 3).

**Table 3:** Example of situation one, where the facilitator uses open-ended questions

Line	Speaker	Utterance	Fac conduct	Other actions
1	Fac	If you should have done something different, what would you have done?	Fac looks at N3, points at N3 with the right index finger.	
2	Fac	Is there anything you think of in that regard?	Still looks at N3.	
3	N3	Provide clearer information when speaking to the doctor.	Fac nods while looking at N3.	N3 nods when looking at Fac.
4	Fac	What did you consider unclear?	Smiles briefly, while maintaining a gaze at N3.	N3 looks at Fac and laughs.
5	N3	I was unsure when I followed ISBAR. about the order…		

Here, the facilitator asks several open-ended questions to prompt N3 to reflect on how she would have handled the situation if she had called the doctor again. By using open-ended questions, the facilitator also encourages N2 to reflect on what happened during the situation.

#### Advocacy inquiry

Advocacy inquiry is a questioning technique where facilitators combine observations and opinions with a question [32]. By sharing their perspective, facilitators invite students to articulate their reasoning and mental models, the beliefs and assumptions that shape their actions [2]. This approach promotes deeper learning by uncovering the rationale behind learners’ decisions, aiming for mutual understanding rather than simply correcting behavior [23]. The process involves presenting an observed action, explaining its significance, and asking students to share their thoughts. A key feature of advocacy inquiry is transparency: both facilitators and students openly reveal their thinking [36].

One such situation, in which the health of a 76-year-old man deteriorated due to post-operative bleeding, aptly illustrates the facilitator’s use of advocacy inquiry (see Supplementary file 1, Situation 2, for details) (Table 4).

**Table 4:** Example of situation two, where the facilitator uses adcocacy inquiry

Line	Speaker	Utterance	Fac conduct	Other actions
16	Fac	If you have a heart rate of 129, you will feel it here.	Fac puts the left hand over the chest while looking at N1.	
17	Fac	For this patient, 129 is relatively high.	Rest the right hand on the documents in the lap while still looking at N1.	
18	Fac	What do you consider the standard values?	Holds eyes on N1.	
19	N1	Eh, 70 or 80?	Fac nods several times.	N1 is a bit hesitant.

In this situation, the facilitator first makes an objective statement, noting that the heart rate is 129. They then repeat the message with a subjective interpretation, commenting that 129 is relatively high. Following this, the facilitator poses an open-ended question, which N1 answers hesitantly.

#### Circular questions

Circular questions invite a third party to describe the interaction between two other individuals in the presence of both [36]. This technique helps facilitators explore interdependent behaviours and recurring patterns within a team. By using circular questions, facilitators can generate insights, encourage perspective-taking, reframe problems, and situate actions in a relational context [32].

A situation that reflects the facilitator’s use of circular questions is when the facilitator directs observer 3 about how she comprehended how N1 and N2 handled the double-check when administering the glucose injection to the women suffering from hypoglycemia (see Supplementary file 1, situation 3, for details) (Table 5).

**Table 5:** Example of situation three, where the facilitator uses circular questions

Line	Speaker	Utterance	Fac conduct	Other actions
18	Fac	Do you have any comments on the double-check?	Looks around at the the students on the left side of the room, making hand gestures that move forth and back with fingers spread.	Some students look down.
		(2 seconds of silence)		
19	Fac	Was everything executed correctly, or are there areas for improvement?	Gaze stops at O3.	
		Two seconds of silence.	Nods repeatedly.	
20	O3	I felt their double-check was short or inadequate.	Fac looks at O3 while nodding.	
21	Fac	Mm, mm.	Fac smiles a bit, and touches the chin with left-hand fingers.	
22	O3	It was a bad double-check.		
23	O3	Nurse 1 waved the syringe and asked if it was correct, and Nurse 2 briefly glanced and said it was okay. It seemed a bit haphazard to me.	Still looks at O3.	
24	Fac	Yes, yes. How did you experience the double-check? Did you get a double-check?	Turns toward N2, looks directly at her while pointing with the index finger of the left hand.	

Here, the facilitator briefly looks at the observers, then focuses on O3 and asks an open-ended question about N1 and N2’s double-check. When O3 hesitates, the facilitator follows up with a closed question. O3 responds that she thought the double-check was insufficient and explains how it was performed. The facilitator then asks N2 to share her experience with the double-check, as she was the one responsible for carrying it out.

### Positive feedback

The facilitators frequently used positive feedback in the four situations, primarily when students reflected and proposed correct answers to their questions. Verbal affirmations, such as supportive responses to student suggestions were often reinforced with a smile, a nod, and a forward-leaning posture. A supportive facilitator fosters inclusivity and empowerment, actively encouraging student participation [37]. Positive feedback and encouragement have been shown to boost learners’ motivation and confidence, making them more receptive to feedback and more likely to engage with the simulation [38].

A telling example of positive feedback can be found in the case of the deteriorating 76-year-old man, in which the facilitator invites the students to clarify which measures fall under C (Supplementary file 1, case 2) (Table 6).

**Table 6:** Example of situation two, where the facilitator provides positive feedback

Line	Speaker	Utterance	Fac conduct	Other actions
32	Fac	What other measures should be taken under circulation? 2 s of silence.	Fac looks at N1, then at N2.	
33	O6	Blood pressure?	The facilitator wraps their right hand around their left hand in front of their body, looking at the observers seated on the right side. The gaze stops at O6.	O6 hesitates when suggesting this alternative.
34	Fac	Blood pressure, yes, that is good.	Fac looks at the observers seated on the right side of the room.	Some students laugh.
35	Fac	Yes, blood pressure is correct.	Fac laughs a little Looks around at the other students.	
36	Fac	Now, we are moving in the right direction.	Fac laughs a little.	All students laugh.

The facilitator encourages participants to identify additional measures related to circulation. When O6 hesitantly suggests blood pressure, the facilitator responds with positive feedback, noting that the suggestion is correct and that they are now moving in the right direction.

### Hints and cues

Facilitators typically used hints and cues when students gave incorrect answers, hesitated, or did not respond. During debriefing, they employed strategies such as cues and hints to encourage independent problem-solving [39]. Hints or cues are triggers that direct attention to key or secondary information [11]. According to the Healthcare Simulation Standards of Best Practice, facilitation involves delivering predetermined or spontaneous cues to help participants achieve learning outcomes [40]. While these standards recommend both types of cues, they do not specify how they are applied during debriefing.

The following example shows a facilitator using a hint to guide students in systematically applying the ABCDE assessment framework when managing a patient with COPD deterioration (see Supplementary file 1, case 4, for details) (Table 7).

**Table 7:** Example of situation four, where the facilitator uses a cue to help the students

Line	Speaker	Utterance	Fac conduct	Other actions
33	Fac	You explicitly suggested conservative management by placing the patient in an upright, forward‑leaning position and encouraging pursed‑lip breathing. You also highlighted something very important: focusing on expiration.	Fac sits toward O1 with a glance at her. Demonstrates how the patient might sit in a forward-leaning posture, Shape the fingers of the left hand into a pursed-lipped gesture, holding them in front of the mouth to illustrate.	The other students pay attention to fac explanation.
34	Fac	Regarding patients with COPD, is inspiration or expiration the most prominent problem?		
35	O3	Expiration.	Fac nods.	

The facilitator commends Observer 1 (O1) for suggesting a conservative treatment approach and offers a hint about the patient’s primary concern: expiration. Immediately afterwards, the facilitator asks the students whether inspiration or expiration is the main problem, which O3 correctly answer.

### Suppressions

In the sequences we studied, facilitators employed suppression when students gave incorrect answers or demonstrated misunderstandings. Suppression took various forms, including brief moments of silence, the absence of verbal response, or shifts in nonverbal communication such as facial expressions, gaze, and body posture. We interpreted and classified these behaviors as suppression, evident in facilitators’ tendency to withhold verbal feedback or rely on nonverbal cues following incorrect answers, whereas correct answers elicited explicit verbal confirmation.

An example of suppression is shown when the facilitator invites the student to explain how they considered prioritising the actions for the patient with COPD exacerbation (see Supplementary file 1, situation 4, for details) (Table 8).

**Table 8:** Example of situation four, where the facilitator uses suppression

Line	Speaker	Utterance	Fac conduct	Other actions
57	Fac	Try to follow the oxygen from the nose down to the lungs, around the body, and into the brain, where it reaches the respiratory center.	Fac looks at N2.	
58	Fac	What could have happened if the patient received too much oxygen?	Fac does not answer N1’s question.	
59	N1	Does the body produce toxins, making the patient more intoxicated, like it would poison the patient?	Gaze fixed at N2, Fac nods.	
60	N2	I believe the level of CO₂ increases, and these patients are not able to breathe out CO₂ like normal people do.	Fac looks at the students	
61	O3	If you get more oxygen, there will be more CO₂ so that you will have a problem breathing out again.	Fac do not respond to this comment	
62	Fac	Follow the oxygen around the body; how does the respiration center react when it gets an extra liter?	Repeats the movement with the right hand (following the trajectory of oxygen from the nose to the brain). Looks directly at N1and N2.	

N1 tries to follow the instructor’s request to trace the path of oxygen from the mouth, down into the lungs, into the blood and finally to the respiratory center of the brain. When N1 struggles to explain, the instructor remains silent. N2 then attempts to give an answer, which is also incorrect, and the instructor waits a few more seconds before repeating the suggestion about following the path of oxygen through the body to the group of students. In this case, we can see that the facilitator draws on two techniques to achieve suppression: silence and repeating a previous question.

### Summaries

The final communication element we observed was the facilitators’ use of summaries, typically after a topic had been thoroughly discussed. These summaries marked transitions between learning objectives, reinforced correct procedures, and referenced relevant guidelines or frameworks. Although most facilitators ended with a section where students shared their take-home messages, they occasionally found it appropriate to summarise during the debriefing to maintain flow and clarity.

An apt illustration of how summaries are used emerged when the facilitator summarised what happens when N1 and N2 administered intravenous glucose to the young woman with hypoglycemia (Supplementary file 1, case 3) (Table 9).

**Table 9:** Example of situation three, where the facilitator uses summaries during debriefing

Line	Speaker	Utterance	Fac conduct	Other actions
36	Fac	This is a realistic situation that you can encounter in the clinic.	Looks around at all the students, moves both hands up and down with fingers spread on the left hand and holding on to a pen in the right hand.	Several students nod.
37	Fac	In stressful situations, mistakes can happen because we are under stress.		
38	Fac	We want to do things fast, and just then, it is crucial to pause, breathe for 10 seconds, and double-check.	Fac looks alternates between the students.	
39	Fac	Verify the patient’s medication chart, identify the patient, and provide the medication details, including strength and dosage.	Turns the head and points toward the medication chart, which is placed on the table to the facilitator’s right.	
40	Fac	The person who double-checks must also confirm these details.	Touches the right-hand fingers with the left hand (like counting).	
41	Fac	This scenario reflects everyday clinical situations and potential mistakes.	Touches the right-hand fingers with the left hand.	Some students nod.

To conclude the discussion on safe medication handling, the facilitator summarises key points before moving to the next learning objective. The summary emphasises that students are likely to encounter similar situations in clinical practice and highlights essential steps for administering and double-checking medications.

## Discussion

In our analysis, we identified five communication elements employed by facilitators: inquiries, positive feedback, hints/cues, suppressions, and summaries. Because the first three elements are well-established in the debriefing literature, we will address them briefly before turning to a more in-depth discussion of the less familiar concepts of suppression and summaries.

Facilitators often experience tension during debriefings [29, 41]. They must balance fostering psychological safety and encouraging reflection with the challenge of providing honest feedback without harming social relationships, a dilemma referred to in the introduction as the ‘task versus relationship’ conflict [4, 29, 32]. In this context, we will discuss the five identified communication elements that facilitators used to elicit reflection through meaningful discussion and student engagement: inquiries, positive feedback, hints/cues, suppressions, and summaries.

### Use of inquiries

We can assume that the facilitators in the sequences described above were interested in eliciting students’ reflections by fostering discussions. In the first three illustrative excerpts, the facilitators used open-ended questions when addressing the students in situation 1 [32], and we also identified elements of advocacy inquiries and circular questions in Tables 4 and 5 [11]. Open-ended questions were often used when the facilitator initiated the debriefing.

Previous research has highlighted open-ended questions as an effective practice for promoting student reflection during debriefing [32, 36], with Fanning and Gaba identifying their use as a key element of successful debriefing [7]. Similarly, Pannekoeke et al. reported that ongoing training and peer feedback increased facilitators’ awareness about the value of open-ended questions and encouraged students to elaborate on their answers, thereby improving facilitators’ ability to identify knowledge gaps and foster reflection [42]. Mohammad used discourse analysis to find that facilitators frequently employed “wh” questions (who, what, why, where, how) to engage students and observers [43]. She concluded that open-ended questions effectively sustained discussions, though prolonged use could have led to longer, more profound reflections. Consistent with these findings and established debriefing frameworks [22, 36, 44, 45], our study showed that facilitators who successfully elicited student reflection and discussion relied heavily on open-ended questions.

Another debriefing technique we identified elements from was advocacy inquiry, which was prominent in the case of the 76-year-old man with postoperative bleeding. In an interview study with expert facilitators, Krogh et al. found that facilitators consciously employ strategies, such as advocacy–inquiry questions, in debriefings [45]. Similarly, Timmis and Speirs surveyed final-year medical students who had participated in debriefings based on a debriefing framework which included the advocacy–inquiry method (AIM). The study found that the majority felt this method effectively encouraged them to reflect on and challenge their thoughts, attitudes, and beliefs [46]. Our research extends these findings by demonstrating that facilitators who successfully promoted student reflection and communication applied advocacy–inquiry techniques in practical situations, thus aligning their approach with structured debriefing frameworks [22, 39, 40].

A third communication style within the family of inquiries was the facilitators’ use of circular questions. In our research, circular questions often occurred when facilitators consulted a third party, frequently an observer, to give their perspective on the relationship between two active students, which is the primary purpose of a circular question [24, 30]. This could be seen in the excerpts regarding observer 3, who was asked how she considered the double-check done by N1 and N2 to the woman with hypoglycemia. Berger-Estilia found that using circular questions and increasing participant input significantly enhanced learning outcomes [47]. This approach not only broadened participation but also fostered shared reflection. While previous research has highlighted the role of circular questions in structured debriefing frameworks [30], our findings show how facilitators apply them in practice to maintain engagement and integrate diverse perspectives. This supports the argument that successful facilitation involves a dynamic interplay between pedagogical intent and moment-to-moment responsiveness. Techniques such as circular and open-ended questions appear to be particularly useful for promoting self-reflection [24, 30].

### Positive feedback

Positive feedback plays a key role in simulation-based education by fostering psychological safety and supporting student engagement [48, 49]. Studies show that this approach helps students feel more comfortable reflecting on their performance and reduces self-criticism, while affirming feedback can enhance confidence [50, 51]. Positive feedback may evoke pleasant emotional responses in participants, but an individual may find corrective feedback more useful [52]. McQueen et al. found that facilitators experienced it was easy to give positive feedback, while they found it challenging to provide negative feedback [53]. Similarly, Lervik et al. describe how experienced nurse facilitators believe that all students should receive positive feedback to achieve learning outcomes [25]. However, relying solely on positive feedback without corrective input may limit learning opportunities [54]. In our material, facilitators consistently used positive feedback during debriefings, aligning with frameworks such as DASH, Plus–Delta, Advocacy-Inquiry, and PEARLS [22, 55–57]. Although we did not assess the emotional impact of these practices, and therefore cannot make claims about their affective outcomes, our analysis demonstrates how they maintain engagement and promote reflection in naturalistic situations. Cues and hints

### Cues and hints

Although research on how facilitators use hints and cues during debriefings remains limited, especially when addressing mistakes and giving feedback, some studies have examined the effects of different debriefing strategies on promoting student reflection and learning [41, 58]. Cues function as instructional support, helping learners regain orientation within a scenario or progress to the next step [59]. Comparable supports, such as hints, prompts, help features, feedback, and coaching, differ in intensity, and these variations often distinguish different instructional approaches [60, 61]. Analysing videos of routine formal feedback in clinical contexts across diverse health professions, Johnson et al. found that facilitators used various practices to position themselves as allies by showing helpfulness, care, and humility, all while students closely monitor the facilitators’ cues during debriefings [62]. Similarly, Solli et al. found that students considered the timing of cues and hints to be essential, enabling them to solve problems independently before facilitators intervened [63]. Our observations resonate with these findings: we interpreted the facilitators’ use of hints and cues as a form of instructional support, aiming to steer students towards correct responses, while preserving a sense of autonomy and mastery.

### Suppressions

Facilitators occasionally used suppressive strategies when students showed flawed reasoning, made mistakes, or behaved inappropriately, a practice rarely noted in debriefing literature. These suppressions included brief silences, nonverbal cues, withholding responses, or repeating questions. Corrections and advice are interactionally delicate and can provoke tension [64]. Such practices warrant caution as they may undermine psychological safety and hinder learning [62]. Meaningful debriefing relies on an environment that fosters reflection and dialogue [65, 66] which further requires skilled facilitation [63, 67]. Well-timed and calibrated silences coupled with minimal responses can create space for articulation and self-reflection [7, 68].

Havery found that facilitators who used silence, active listening, and minimal cues (e.g., “mm” and “gee”) encouraged greater student participation [69]. Silence can allow cognitive processing. In dyadic exchanges, participants typically pause before responding [70]. Waznonis’ mixed-method study found that experienced facilitators often used silence to foster engagement, whereas novices struggled to tolerate it, sometimes misinterpreting silence as confusion [71]. Conversely, White et al. found that few faculty members intentionally used silence, and when they did, it was often ineffective because facilitators answered the reflective questions instead of the students [72]. While remaining silent can be challenging, facilitators may need to develop a tolerance for such pauses [41]. Consistent with prior research, our findings indicate that silence and minimal responses frequently encouraged students to elaborate and reflect more deeply.

### Summarising

Our analysis shows that facilitators often use summaries during debriefings, typically after a topic has been fully explored. For example, after a discussion on glucose medication management, the facilitator summarised the correct approach to preventing mistakes and adverse events. The simulation literature describes summaries in various ways [41]. Coggins et al. (2020) observed interdisciplinary debriefings using the “S.T.O.P.” model, which starts with a case summary, successes, areas for improvement, and action points [65]. Similarly, Macdiarmid et al. (2020) found that experienced facilitators often begin with an event summary to help learners move past emotional intensity and focus on learning. In addition, they sometimes conclude with key takeaways [41].

While practices vary, summaries appear to facilitate transitions between discussion points and reinforce learning. They are widely regarded as best practice in simulation-based education, consolidating learning, clarifying objectives, and providing psychological closure [45]. Structured models such as the three-phase approach (Reaction–Analysis–Summary), PEARLS, and the Healthcare Simulation Standards of Best Practice consistently include a summary phase to ensure actionable insights and emotional resolution [12, 22, 73]. According to PEARLS, summaries can be learner-driven, with students stating their main takeaways, or facilitator-led, with the facilitator providing a concise review of key points [22]. Summaries made during the debriefing did not replace the final summary, in which students were asked to share their take-home message. While most facilitators conducted this summary as part of the debriefing, our data suggests that facilitators occasionally omit it.

### Strengths and limitations

This study has some methodological strengths that enhance the credibility of its findings. Approximately 70 h of video data from thirty-six simulation sessions provided a rich dataset for analyzing facilitator–student interactions and validating observed patterns. The first author’s presence during data collection and contemporaneous field notes enabled early familiarization and contextual insight. Her professional background as an intensive care nurse and experienced simulation facilitator further informed a nuanced analysis of how facilitators addressed mistakes. A dual analytical approach, combining video analysis with thematic analysis, offered a robust, multifaceted examination of the data.

However, participation was voluntary, which may have led to a self-selecting sample of confident or motivated students, potentially influencing debriefing dynamics. Additionally, the author’s professional background, while valuable for interpretation, introduces potential bias.

## Conclusion

A primary contribution of this study is to identify the specific communication elements that facilitators use to elicit rich student reflection and discussion. These include suppressions and the use of multiple interim summary techniques, which are not well documented as distinct communication elements in the simulation debriefing literature. While current structured debriefing frameworks may feature some aspects of these elements, none appear to integrate the full range identified in our observations. Consequently, future debriefing frameworks could be enhanced by systematically incorporating this broader array of communication strategies. Given the limited research examining the nuanced, in-action techniques facilitators use to stimulate student reflection, further investigation is needed to deepen our understanding of their role in optimizing debriefing practices.

## Supplementary Information

Supplementary Material 1.

## Data Availability

Larger transcripts are provided as additional files accompanying this publication. Other data generated and analysed during the study are available from the corresponding author upon reasonable request, following ethical guidelines and participant confidentiality.

## Abbreviations

COPD: Chronic Obstructive Pulmonary Disease
ABCDE: Airways-Breathing-Circulation-Disability-Exposure
ISBAR: Identification-situation-background-assessment-advice
NEWS: National Early Warning Score
