# Supplementary material for: How facilitators use healthcare students’ mistakes to promote reflections and discussions during simulation debriefings
Source: Adv Simul (Lond). 2026 Feb 5;11:19. doi: 10.1186/s41077-026-00412-3 (PMC12964741; doi:10.1186/s41077-026-00412-3)
Supplement: Supplementary file 1 — Supplementary Material 1. [file 41077_2026_412_MOESM1_ESM.docx]

**Supplementary file 1: Extended transcripts**

**Situation 1 - Respiration**

**
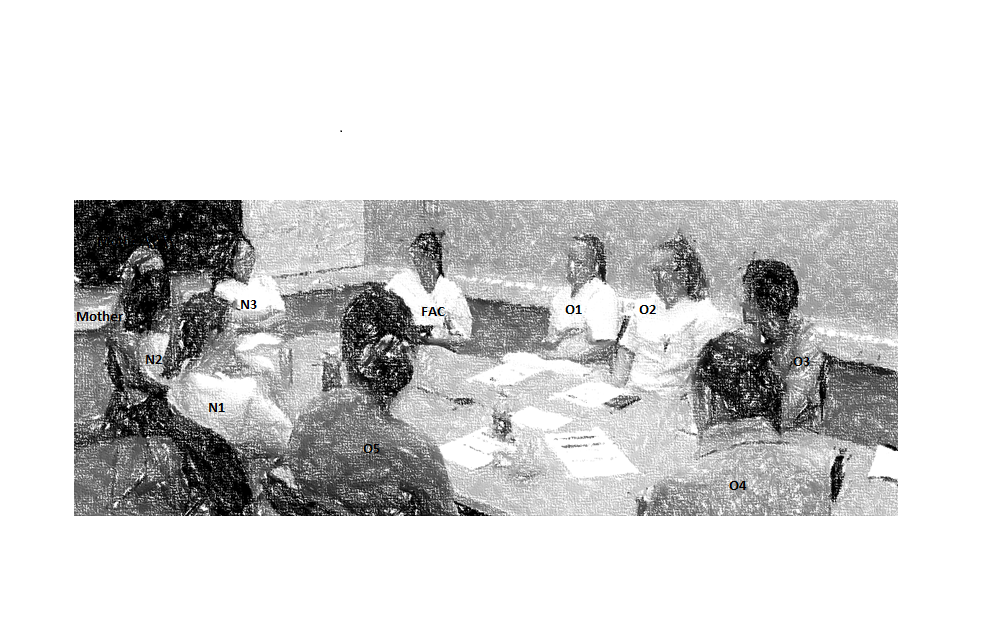
**

Three nursing students played the roles of N1, N2, and N3, while a fourth student portrayed the patient’s anxious mother during simulation training on a baby with respiration challenges. Five other students served as observers (O1-O5) and wrote notes regarding specific learning objectives. The learning objective in this situation was to use the ISBAR communication tool. The operator in an adjacent room answered the phone as a doctor when N2 called. In this scenario, a five-week-old baby was admitted to the children’s ward due to breathing problems. The baby’s tentative diagnosis was bronchiolitis. During the scenario, N3 called the doctor when the baby’s breathing deteriorated. During the phone call, N3 did not follow the steps in the ISBAR communication tool. In the following excerpt, the team discusses the learning objective ISBAR.

The facilitator initiates the discussion seven minutes into the debriefing.

| **Line** | **Speaker** | **Utterance** | **Facilitator’s conduct** | **Other action** |
| --- | --- | --- | --- | --- |
| 1  2 | Fac  Fac | If you should have done something different, what would you have done?  Is there anything you think of in that regard? | Fac looks at N3, points at N3 with the right index finger.  Still looks at N3. |  |
| 3 | N3 | Provide clearer information to the doctor. | Fac nods while looking at N3. | N3 nods when looking at Fac. |
| 4 | Fac | What did you consider was not clear? | Smiles briefly, while maintaining a gaze at N3. |  |
| 5 | N3 | I was unsure when I followed ISBAR.. about the order… |  | N2 laughs. |
| 6 | Fac | Oh, yes | Smiles a little bit. |  |
| 7 | N3 | I was so stressed. | Smiles at N3. | N3 looks at Fac and loughs. |
| 8 | Fac | So, you were stressed? | Nods and smiles. |  |
| 9 | N3 | But it turned out okay, after all? | Nods more and smiles. | N3 laugh. |
| 10  11  12 | Fac  Fac | Yes?  Was it something special you thought that you should have said before, or sooner?  Was there something you did not mention, or omit? | The gaze remains locked on N2, unwavering. The left hand clutches a stack of papers, firm but relaxed. Meanwhile, the right hand moves in slow, circulating gestures, drawing invisible patterns in the air, emphasizing each word with rhythmic motion. Leans forward against N3. |  |
| 13 | N3 | I said everything, but it was not in the right order. | Still, looks at N2, nods a few times. |  |
| 14  15  16  17 | Fac  Fac    Fac  Fac | Not in the correct order, no.  What consequences can it have if you don’t follow these orders?  Why is there a systematic issue with the ISBAR tool?  Does it mean this much, or what do these orders mean? | Moves the head slightly to the right side, leaning back in the chair, and looks at the other students; the right hand moves in small circular motions in front of the body.  Gaze shifts from N2, briefly to the mother, and further to N3. |  |
|  |  | Three seconds of silence |  |  |
| 18  19 | O4  O4 | To ensure that the one [person] you ask for advice shall have all the available information they need.  Because if you say, “I am me, and I need advice,” the doctor hasn't got any information about the patient | Gaze settles on O4. A series of deliberate nods follows, slow, measured, affirming. Each nod appears to signal agreement, acknowledgement, or silent confirmation. | O4 points toward the papers on the table in front of the body. |
| 20  21 | N3  N3 | Then I asked the doctor, What am I going to do?  Then he said, “Connect 5 liters of oxygen after inhalation”, and I did it. | Turns head against N2, nods and smiles  Nods several times. | Still laughing a bit. |
| 22 | O4 | The receiver is also used to receive the information in that order. | Looks at O4, nods several times. |  |
| 23  24  25  26 | Fac  Fac  Fac  Fac | Yes, but even if you did not ask for it, you got the measures.  It is not unusual that the measures come before..  One tells about the situation, and then it comes:  “Okay, you do it like this”.  It's not often you have to ask: what shall I do, because it comes, right? | Turns head toward N3again, looks at N3, and hand gestures in circular movements with palm facing outward.  Hands in circular movements in front of the body. | A short pause between sentences 24 and 25.  Several students nodded. |
| 27 | N3 | I was satisfied that I confirmed the doctor's orders | Looks at N3. | N3 is serious when talking. |
| 28  29  30 | Fac  Fac  Fac | Yes, you confirmed.  Did you hear that, too?  Hm, did any of you others hear that phone call, or were you..? | Fac nods while looking at N3, then turns her head to the left side, looking at the observers.  Turns toward the observers on the left side while looking at them.  Then, fac turns gaze to the active students on the right-hand side. | Some observers nod.  3 seconds of silence. |
| 31  32 | N1  N1 | I heard the end of it.  I heard she said 5 liters. | Looks at N1, nods several times, then looks at the papers in her lap. | Talks in a low voice. |
| 33 | Fac | Did you tell him where you were located? | Looks at N3, raises the hand towards N3. |  |
| 34 | N3 | Yes. I told the baby's name and age of the baby. | Looks directly at N3, nods several times. |  |
| 35 | Fac | Yes, yes, yes | Nods several times. |  |
| 36 | N3 | And that she was admitted to the children's ward | Looks directly at N3. |  |
| 37  38 | Fac  Fac | Yes  Did you tell him [the doctor]about the diagnosis? | Nods again. |  |
| 39 | N3 | Yes. |  | N2 hesitates. |
| 40  41 | Fac | Hm….  I think it came a little too late. | Points with their fingers like steps in the air in front of the chest. Gaze still on N3. |  |
| 42  43 | N3  N3 | Yes, it did..  Because the doctor had to ask what was wrong with that baby. | Smiles and points with the right index finger toward N3. | N2 laughs when answering. |
| 44  45  46  47  48  49 | Fac  Fac  Fac  Fac  Fac  Fac | Yes, because they have a tentative diagnosis when admitted, right?  And then it is, okay, a nurse from the children’s department:  “I have a five-week-old baby with a tentative diagnosis of bronchiolitis and am admitted for that.”  Then you have the basis for the diagnosis, and you can add all the symptoms that occur because of this diagnosis.  However, you handled it in the end; we heard you say bronchiolitis eventually.  Do you agree? | Turns the gaze to the students on the left-hand side.  Gaze alternate from student to student.  Moves the right hand in small circular movements.  Holds a pen in the right hand, knocking the pen rhythmically in the air when stating the opinion. Moves both hands in more prominent, circulating movements before looking back at N2**.** |  |
| 50 | N3 | Yes, I did. |  | N2 still laughs. |
| 51  52  53 | Fac  Fac  Fac | This is a matter of training.  This systematics, right, when you speak to someone.  Just repeat for yourselves also, | Puts down the pen,  Hand gestures in circulating movements. |  |

**Situation 2 – Postoperative bleeding**


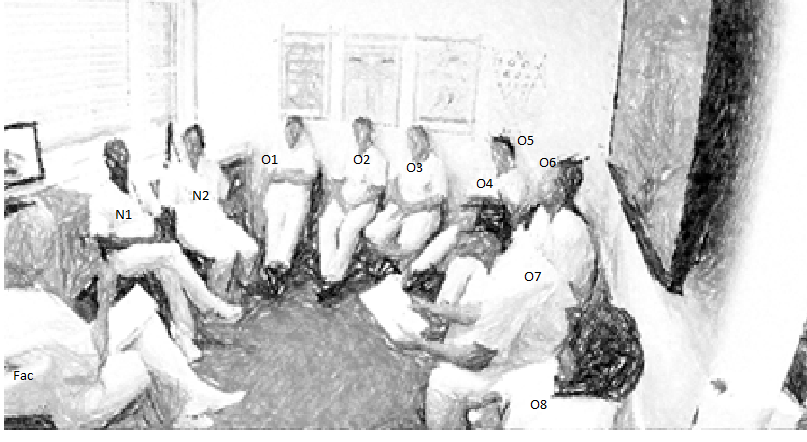


Two nursing students play the roles of N1 and N2 during a simulation-based training on postoperative bleeding, while eight other students serve as observers (O1-O8) and wrote notes regarding specific learning objectives. The scenario was a 76-year-old man who had undergone an operation on his left hip the night before. His condition was deteriorating with high respiration and heart rate, low SpO2, and low blood pressure. The patient was pale and complained about pain in the operating area. When N1 and N2 entered the room, they measured the patient’s vital signs, but they did not notice the patient’s deteriorating condition due to post-operative bleeding. In the following excerpt, the facilitator and students reflect on and discuss actions and behaviors related to the learning objective, observing and handling the ABCDE procedure. In the excerpt below, the group has completed their discussion on airways and breathing and is now about to discuss the issue of circulation.

The facilitator initiates this discussion 14 minutes into the debriefing.

| **Line** | **Speaker** | **Utterance** | **Facilitator’s conduct** | **Other actions** |
| --- | --- | --- | --- | --- |
| 1 | Fac | What comes under circulation? | Fac looks at Nurse 1 while nodding, then leans her head back, keeping her gaze on Nurse 1. Fac glances down at a paper note in their lap before looking back at Nurse 1. Fac sits upright in the chair, left leg crossed over the right, hands held together with palms facing each other in front of the chest, looking around at all the students. |  |
| 2 | N2 | Heartrate? | Looks at N2 and nods. | N2 hesitates when suggesting this. |
| 3 | Fac | The value is 129 | Still looks at N2. |  |
| 4 | N1 | That’s high | Fac glance shifts to N1. | N1 uses a low voice. |
| 5  6 | Fac  Fac | Do you think it was high?  What would you have expected in this patient?  2 seconds of silence follows. | Fac looks at N1 while nodding, then leans back with their gaze still on Nurse 1. They glance down at the paper on their lap before looking back at Nurse 1. |  |
| 7 | N1 | Maybe it is because the patient has lost a lot of blood? | Fac separates their hands and leans forward toward Nurse 1, placing the left hand on the papers in their lap. | N1 hesitates when suggesting this.  2 seconds of silence follows. |
| 8 | Fac | Yes, he was 76 years old. | Fac looks at N1. |  |
|  |  | Silence for 2 seconds. |  | N1 does not respond. |
| 9  10 | Fac  Fac | He was 76 years old; he had no heart disease.  What would you consider the value then? | Still looks at N1. |  |
| 11 | N1 | The standard values? | Looks at N1. |  |
| 12  13  14  15    16  17 | Fac  Fac  Fac  Fac  Fac    Fac | Yes, you must always compare it to a standard.  Where will you place these values?  You must see it in relation to something.  If you have a heart rate of 129, you will feel it here.  For this patient, 129 is relatively high.  What do you consider standard values? | Fac raises both hands and gestures them sideways a few times, maintaining eye contact with Nurse 1, with her left palm facing outward towards Nurse 1.  Fac puts the left hand over the chest while looking at N1.  Rest the right hand on the documents in the lap while still looking at N1.  Holds eyes on N1. |  |
| 18 | N1 | 70 or 80? | Fac nods several times. | N1 is a bit hesitant. |
| 19 | Fac | And then he has a heart rate of 129  Have you ever experienced a heart rate of 129?  You have all had 129 in heart rate, right? | Fac waves with the left hand in front of the chest while maintaining gaze on the students to the right.  Fac then turns head to look back at Nurse 1, extending left arm towards the student. | All the students nod. |
| 20 | N1 | If you are at the gym, perhaps? | Smiles, still looks at N1. | N1 uses a low voice.  All students laugh. |
| 21 | Fac | Right, and if you have a heart rate of 129, you’ll feel it here. | The facilitator places both hands on their upper chest and neck, tapping with their fingers while looking at the observers seated in front of them. |  |
| 22 | Fac | And he said that he felt his heart beating, right? | Looks at all the students, then back at N1, before turning to the other students again. | Some students nod. |
| 23  24    25 | Fac  Fac  Fac | That means a heart rate of 129 is relatively high.  What does this mean?    What can be the reason for the high heart rate in this patient?  Three seconds of silence. | Fac looks at N1.  Still looks at N1.  Fac looks at the students seated on the right side of the room. Tap upper chest and neck, alternating between right and left fingertips.  Gaze shifts back to N1, and then N2. |  |
| 26 | O8 | Can it be hypovolemia? | Fac turns toward Observer 8, nods several times, holding hands together in front of the chest. |  |
| 27 | Fac | Yes, and how can you know if it is hypovolemia? | Fac looks directly at Observer 8 before shifting their gaze toward Observers 5 and 6.  Fac then alternates gaze among all the observers and extends the right arm toward them, pointing with the index finger toward Observers 5, 6, 7, and 8**.** |  |
| 28 | O4 | Bleeding? | Fac looks at Observer 4, moves hands slightly in front of chest before clasping left hand and wraps the right hand around the left hand. |  |
| 29 | Fac | Yes, but how can we know if it is bleeding? | Fac shifts gaze back to Observers 5 and 6, motioning the hand forward and backwards. |  |
| 30 | O5 | C? | Fac shifts gaze toward Observer 4. |  |
| 31  32 | Fac  Fac | Yes, we are handling the C now.  What other measures should be taken under circulation? | Fac looks at the students on the right-hand side, then shifts gaze to O5, holding hands together.  Fac looks at N1, then  at N2. |  |
| 33 | O6 | 2 seconds of silence  Blood pressure? | The facilitator wraps their right hand around their left hand in front of their body, looking at the observers seated on the right side. The gaze stops at O6. | O6 hesitates when suggesting this alternative. |
| 34  35  36 | Fac  Fac  Fac | Blood pressure, yes, that is good.  Yes, Blood pressure is correct.  Now, we are moving in the right direction. | Fac looks at the observers seated on the right side of the room.  Looks around at the other students.  Fac laughs a little. | All students laugh. |
| 37 | Fac | If you add his symptoms with a high respiration rate, high heart rate, low blood pressure, low oxygen saturation, low temperature, pain and paleness, then you have all the signs of bleeding. | Gaze shifts from student to student. Touches one finger at the time on the right hand with left-hand fingers (counting motion). | Most students looks at the Fac. |

**Situation 3 - Hypoglycemia**


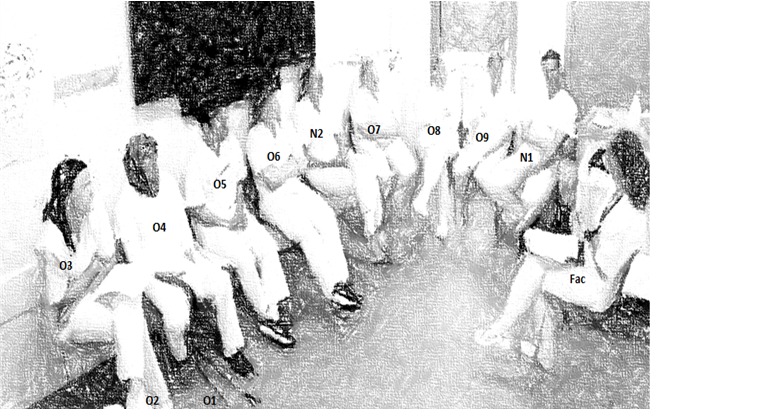


Two nursing students played the roles of N1 and N2 during a simulation training exercise involving a patient with hypoglycemia. A third student, the patient’s friend, accompanied her when she was admitted to the hospital this same morning. The operator in the adjacent room answers the call as a doctor when N1 called. Nine other students served as observers (O1-O9) and wrote notes on specific learning objectives. The learning objective in this situation was safe medication handling. The scenario was a young woman who had recently been diagnosed with type 1 diabetes. When she arrived at the hospital this morning, her blood sugar was low at 1,8 mmol/l, and after the patient refused to drink or eat, she lost consciousness. N1 called the doctor who prescribed intravenous glucose for the patient, but N1 and N2 did not handle the medication management in adherence to current guidelines. In the following excerpt, the facilitator and the students discuss the learning objective of safe medication handling.

The facilitator initiated the discussion 27 minutes into the debriefing as follows.

| **Line** | **Speaker** | **Utterance** | **Facilitator’s conduct** | **Other action** |
| --- | --- | --- | --- | --- |
| 1  2 | Fac  Fac | Now, we will discuss medication handling.  Then, was it you who should observe that? | Fac sits on a chair with the left foot crossed over the right foot. Papers on the lap.  The facilitator looks up from the notes as they sort them. Looks toward O8 and O9. |  |
| 3  4  5  6 | O9  O9  O9  O9 | Yes, it was us.  Nurse 1 checked the patient's identification and then;  It was the correct medication, but I was unsure if you checked that the medication and the prescribed medication were the same.  It happened a bit fast, so we could not see if you... | Fac maintains eye contact with O9 while O9 speaks, nods in response, and rests both hands on the papers, left hand placed over the right. | O9 looks at N1 when talking.  O9 hesitates during the last sentence, “We did not make it to see if you”. |
| 7 | O8 | Did you look at the medication chart? | Still looks in the direction of N1, O8, and O9. | O8 looks towards N1. |
| 8 | N1 | Yes, when I was on the phone with the doctor. |  |  |
| 9  10 | O9 | Yes, it was the correct dose.  You said it when the double-check..., just in time. | Fac shifts gaze from O8 and O9 to the papers on their lap, then redirects attention back to the observers, O8 and O9. | O9 laughs, and several other students start laughing. |
| 11 | N1 | After being reminded of it, yes. | Fac holds gaze on N1. | N1 adopts an ironic tone. |
| 12  13  14 | O9  O9  O9 | You checked that it was correct, yes, right time, eh  And the right way, you gave it in the PVK.  But we did not notice if there was any flushing | Fac looks down at the papers on their lap and points to a specific line using the index finger of the right hand. Fac nods in response when O8 and O9 state they did not notice any flushing. |  |
| 15 | N1 | That’s right, I did not do that. | Nods while gaze is on N1. |  |
| 16 | O8 | Before and after the injection. | Still looks in the same direction while nodding. |  |
| 17 | O9 | Yes, before and after, yes. | Turns the head towards the students on the other side of the room, points with the index finger while looking alternately at the students. |  |
| 18  19 | Fac  Fac | Do you have any comments on the double-check?  Was everything executed correctly, or are there areas for improvement? | Looks around at the students on the left side of the room, making hand gestures that move forth and back with fingers spread.  Gaze stops at O3. | Several students look down |
|  |  | Two seconds of silence |  |  |
| 20 | O3 | I felt their double-check was short or inadequate. | Fac looks at O3 while nodding. Smiles a bit and touches the chin with the left-hand fingers. | The student hesitates. |
| 21 | Fac | Mm, mmm. | Nods, remaining gaze at O3. |  |
| 22  23  24 | O3  O3  O3 | It was a bad double-check.  Nurse 1 waved the syringe and asked if it was correct, and Nurse 2 briefly glanced and said it was okay.  It seemed a bit haphazard to me. | Still looks at O3. |  |
| 25  26  27 | Fac  Fac  Fac | Yes, yes.  How did you experience the double-check?  Did you get a double-check? | Turns toward N2, looks directly at N2 while pointing at N2 with the index finger of the left hand. |  |
| 28  29 | N2  N2 | First, Nurse 1 said: “I inject this, and I answered okay, and then they said: What is this?”  Then I looked at it and noticed it was 10 ml, but it had been handled hastily. | Still looks at N2, touching the chin with the left hand while the right hand is resting on the papers on the lap. |  |
| 30 | Fac | But how did you know what should be injected? | Still looks at N2 while touching the neck with the left hand. |  |
| 31 | N2 | I heard the doctor order it when Nurse 1 called her. |  |  |
| 32 | Fac | Yea? | Continues to look at N2. |  |
| 33 | O1 | I think they should have checked the medication chart before injecting the medication. | Turns the head against O1, nods, and smiles briefly. |  |
| 34  35 | Fac  Fac | Mm, yes, yes, mm  And just in situations like this, mistakes happen | Turns against the students on the right side again, then looks at all the students while lifting hands in front of the chest with palms down and fingers spread.  Fac looks around at the students in front of them. | All students looks at Fac. Several students’ nods. |
| 36  37  38  39  40  41  42 | Fac  Fac  Fac  Fac  Fac  Fac  Fac | This is a realistic situation that you can encounter in the clinic.  In stressful situations, mistakes can occur because we are under stress.  We want to do things fast, and just then, it is crucial to pause, breathe for 10 seconds, and double-check.  Verify the patient’s medication chart, identify the patient, and provide the medication details, including the strength and dosage.  The person who double-checks must also confirm these details.  This scenario reflects everyday clinical situations and potential mistakes.  That’s why we used this scenario: you can practice this and be aware of this issue. | Looks around at all the students, moves both hands up and down with fingers spread on the left hand and holding on to a pen in the right hand.  Fac looks alternates between the students.  Turns the head and points toward the medication chart, which is placed on the table to the facilitator’s right.  Touches the right-hand fingers with the left hand (like counting).  Touches the left-hand fingers with the right hand (like counting on fingers) while nodding several times.  Fac shifts gaze from student to student. | Several students nod. |

**Situation 4. COPD excacerbation.**


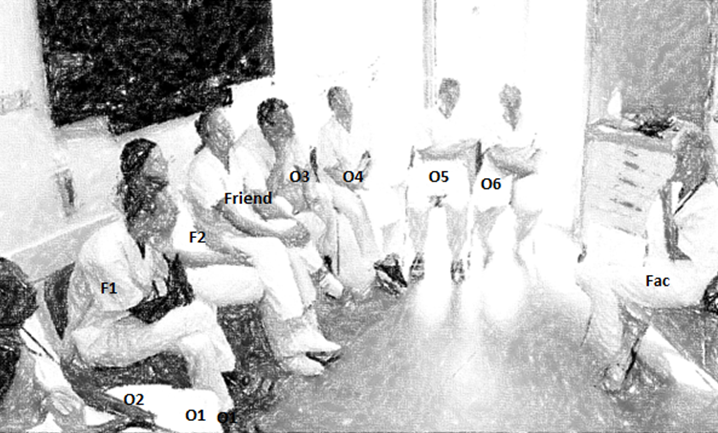


Two nursing students had the roles of N1 and N2 during the simulation training on chronic obstructive pulmonary disease (COPD) exacerbation. A third student played the role of the patient’s worried wife. Six other students served as observers (O1-O6), writing notes regarding specific learning objectives. The scenario was a 70-year-old man with COPD grade 3. He was now admitted due to exacerbation with a high respiration rate of 25/minute and low SpO2 of 85%, and the heart rate was 120/min. According to the medical chart, measures of the other vital signs were acceptable. The patient felt uncomfortable, and his wife was worried; both asked several questions, making it challenging for N1 and N2 to concentrate.

In the following excerpt, the facilitator and the students discuss the learning objective B, which is a component of the airway, breathing, circulation, disability, and exposure (ABCDE) procedure. Seven minutes into the debriefing, the facilitator initiated the learning objective of breathing by asking Nurse 1 the following question.

| **Line** | **Speaker** | **Utterance** | **Facilitator’s conduct** | **Other actions** |
| --- | --- | --- | --- | --- |
| 1  2  3 | Fac  Fac  Fac | Let us talk about the B problem now.  You quickly discovered this was a B-problem, which is good because that is correct.  Who observed the respiration? | Fac faces the students seated in an arc. Fac scans the group, eyes moving from face to face. When O2 begins to speak, Fac leans forward in the chair, visibly engaged.  Looks at N1 and N2 while nodding. | O2 nods and affirms that she was responsible for observing the B-issues. |
| 4 | O2 | That’s me. |  |  |
| 5 | Fac | That’s you. |  |  |
| 6  7  8  9  10  11  12 | O2  O2  O2  O2  02  O2  O2 | Yes, I observed A and B.  A was okey.  You measured the respiration rate, connected the pulse oximetry, and supplied the O2 at 1 liter.  Then I recognised that you reacted to the depth of the respiration; it was a bit superficial and sounded a bit raspy.  Yes, the rhythm also stands here, but I don’t know if they reached that point or what happened.  Yes, and then they helped him into a sitting position and assisted him in clearing his mucus and spitting it out.  You also helped him breathe with narrow lips, enabled him to exhale, that's good. | Fac makes eye contact with O1, nods slightly, with a faint smile.  Fac glances down at the papers (in lap), then looks back at O1, smiling.  Skims the paper again, then gives a subtle nod.  Shifts gaze from O1 to N1 and N2, nodding thoughtfully.  Returns attention to the paper once more. | O1 looks at N1.  Several students laugh.  N1 agrees that this was a good idea by nodding and saying yes. |
| 13  14  15 | Fac  Fac  Fac | We took hold of this B problem because you quickly identified that this was a B problem, which was a deterioration.  What made you prioritize measuring the patient’s SPO2 so quickly?  2 seconds of silence  One measured the SPO2, and the other connected the oxygen supply. | Turns to N1 and N2, gesturing toward them with the left hand.  Sweeps the left hand in the direction where the patient lies, behind the faculty’s back. Then raises the hand, shaping it like a mask, and holds it in front of the face. |  |
| 16 | O4 | Can you please repeat the question? |  | O4 laughs after the utterance. |
| 17  18 | Fac  Fac | When you knew this was a B-problem, and you knew the patient suffered from COPD, and this was a deterioration.  Why didn’t you prioritize that one of you connect the SPO2 measures and one that provided the oxygen supply? | Fixes gaze on N2 while moving the left hand rhythmically up and down, then back and forth in front of the body. |  |
| 19 | N1 | Because we didn’t want to give him too much oxygen? | Fac points toward Nurse 1. Gazes towards N1 with a serious expression. |  |
| 20  21 | Fac  Fac | Yes, oxygen is okay because the patient should have 1 liter of oxygen, so that is okay.  But you were two, so one could have connected the pulse oximetry, and the other could have organized the oxygen. | Shapes a mask with the left hand over the mouth, then moves slowly up and down. Fingers spread open in a deliberate motion, while the right hand holds the paper steady. |  |
| 22 | N1 | Before and after |  | N1 looks at the Fac. |
| 23 | Fac | Yes, you missed the starting value of the saturation and wrote it down only after you started the treatment. In patients with obstructive lung diseases, you should always measure the saturation before connecting the oxygen supplement. | Moves the left hand in a slow, circular motion. Eyes settle on N1 and N2. Raises hand.  Turns to Observer 2. |  |
| 24 | N1 | Oh, yeah |  |  |
| 25 | N2 | We should have measured it first and then supplied the oxygen. | Fac nods. | N2 nods when speaking. |
| 26 | N1 | It doesn’t take long to perform. |  | N1 nods. |
| 27  28  28  30 | Fac  Fac  Fac  Fac | No, not so long.  But you got the question about whether it was with or without oxygen supply, so you could also have connected him immediately.    However, measuring the primary value in patients with pulmonary diseases is a good idea.  That’s priority number 1! | Nods, eyes fixed on Nurse 1. Leans forward in the chair, holding the left hand raised in front of the chest, fingers pressed together.  Turns the head, scanning the students one by one with alternating glances.  Leans back in the chair, fingertips resting on the forehead. Glances down at the papers, then looks up toward O1 and O2. |  |
| 31  32  33 | N1  Fac  Fac | Yes, good.  I want to... it was very nice that you..  You suggested so explicitly the conservative treatment by placing the patient in an upright, forward-leaning position, breathing with narrow lips, and then you said something very good: Expiration! | Fac sits toward O1 with a glance at her.  Demonstrates how the patient might sit in a supine position, back upright and slightly leaning forward. Shape the fingers of the left hand into a narrow-lipped gesture, holding them in front of the mouth to illustrate. | The other students pay attention to the demonstration. |
| 34 | Fac | Regarding patients with COPD, is inspiration or expiration the most prominent problem? | Still looks at O1.  Looks around at the students, then moves the left hand deliberately in front of the chest. Taps the chest with the fingertips, then rotates the hand, palm facing upward in a revealing gesture. |  |
| 35 | O3 | Expiration. | Fac nods. |  |
| 36  37  38  39 | Fac  Fac  Fac  Fac | That’s right.  That’s what you said.  What is happening in the expiration phase?  Why should the patient breathe with narrow lips? | Points toward O1.  Moves the hand steadily up and down in front of the chest, then once again shapes the fingers into narrow lips, holding them in front of the mouth to demonstrate. |  |
| 40 | O1 | We want to create pressure for the patient to breathe in or to exhale. | Fac nods while still pointing at O1. |  |
| 41 | Fac | What are we breathing out? |  |  |
| 42 | O1 | CO2. |  |  |
| 43  44  45  46  47  48  49  50  51  52  53 | Fac  Fac  Fac  Fac  Fac  Fac  Fac  Fac  Fac  Fac  Fac | Yes, good!  It's effective to decrease the amount of CO2.  Because you'll help the body eliminate CO2.  That’s good.  Then you must see that when you started working, the SPO2 increased to 88%.  That is the limit for most of us to be a little confused.  Perhaps a patient with COPD can handle it because they are accustomed to having a low O2 saturation.  But then I am talking about CO2 anesthesia.  There are two dangerous situations where the patient can develop into CO2 anesthesia.  Can you imagine what those mistakes are?  By doing two mistakes? | Fac member shifts slightly in the chair, then raises the right hand to illustrate how the patient exhales CO₂, gesturing outward with a slow, controlled motion.  Lifts the right hand while the paper is in the left hand.  Looks at O1, O2, and N1 and N4 alternately.  Moving the right hand in the direction of N1 and N2.  Looking at the students. | A brief silence follows.  Several students shake their heads. |
| 54  55  56  57  58 | Fac  Fac  Fac  Fac  Fac | You did it right because you gave 1 liter of oxygen  Say you made a mistake and administered 2 liters of oxygen to this patient.  What could have happened to him?  Try to follow the oxygen from the nose down to the lungs, and around the body, and into the brain, where it reaches the respiratory center.  What could have happened if the patient received too much oxygen? | Uses the right hand to trace the path of oxygen, pointing to the nose, then down to the lungs, and motions the bloodstream toward the head. Finally, gestures to the head (likely indicating the location of the brain’s respiratory center). |  |
| 59 | N1 | Does the body produce toxins, and that one is more intoxicated?  2 seconds of silence | Fac do not answer N1’s question.  Faze fixed at N2, Fac nods. |  |
| 60  61 | N2  Fac | I believe the level of CO2 increases, and these patients are not able to breathe out CO2 like ordinary people do.  If you get more oxygen, there will be more CO2 so that you will have a problem breathing out again. | Gaze fixed at N2, does not answer N2, moves the head in small nods, but instead repeats the previous utterance. | N2 looks at Fac. |
| 62 | Fac | Follow the oxygen around the body; how does the respiration center react when it gets an extra liter? | Repeats the movement with the right hand (following the trajectory of oxygen from the nose to the brain). Looks directly at N1and N2. |  |
| 63 | O1 | I think the body gets more oxygen than the lungs can handle, more than normal. |  |  |
| 64 | Fac | Normal? | Looks directly at O1, both hands holding on to the papers. |  |
| 65  66 | Fac  Fac | So, what happens in the respiration center when it registers that there is more oxygen than the usual situation?  What happens with the signals that are related to the mechanism that has with the lung function? | Points to the head with the right hand, then moves it purposefully toward the brain. Scans the room, looking at the students one by one. Extends the right hand outward in a sweeping motion, as if releasing or emphasizing a concept. |  |
| 67 | O3 | Can I say something? | Fac looks at Observer 3. |  |
| 68 | Fac | Yes. | Looks directly at O3 and nods. |  |
| 69  70  71  72 | O5  O5  O5  O5 | You have chemoreceptors in the blood that measure oxygen and CO2 levels.  In healthy lungs, CO2 regulates breathing.  However, in patients with lung diseases, O2 takes over this role.  If these patients receive more oxygen, their respiratory center may reduce breathing, causing CO2 levels to increase. | Fac nods several times. |  |
| 73 | Fac | That’s very good, perfect. | Smiles warmly, nods in agreement, and joins the other students in a round of applause. |  |
